# Designing for a Healthier Makassar, Indonesia: Participatory Systems Mapping

**DOI:** 10.1007/s11524-022-00651-5

**Published:** 2022-07-01

**Authors:** Muh. Afdhal, Andi Alam, Karen Grattan, Bailey Goldman, Ahmad Isa, Amanda Pomeroy–Stevens, Damodar Bachani

**Affiliations:** 1 Building Healthy Cities Project, International Organization for Migration, Jakarta, Indonesia; 2 Building Healthy Cities Project, Engaging Inquiry LLC, Durham, NC USA; 3 Building Healthy Cities Project, JSI Research & Training Institute, Inc, Arlington, VA USA; 4 Building Healthy Cities Project, John Snow India Private Limited, New Delhi, India

**Keywords:** Urban health, Systems thinking, Smart Cities

## Abstract

In Makassar, Indonesia, the USAID-funded Building Healthy Cities (BHC) project engaged 240 multi-sector stakeholders to gather qualitative data across three workshops and two citizen town halls from 2019 to 2021. These data were synthesized with results from BHC’s nine other Makassar activities to build maps of the current system and identify high-impact areas for engagement. Contextual findings showed that Makassar leadership has actively innovated and used new technology to improve the city, resulting in improved connectivity and responsiveness. However, this drive toward innovation has strained existing infrastructure and workforce capacity. When this strain fails to meet promised results, citizens are less likely to engage and support the innovations. This is central to the systems map that BHC developed, and is expanded upon through additional patterns that fall within four main areas: (1) leadership, governance, and financing; (2) infrastructure and workforce; (3) collaboration and data; and (4) community cohesion and awareness. Stakeholders found three key leverage points within this context that, if included in every action, could help overcome barriers. These leverage opportunities are: (1) increasing data-driven decision-making; (2) ensuring equitable policy and leadership; and (3) increasing community participation. By combining key patterns discovered in the Context Map with the leverage opportunities, BHC was able to co-create with stakeholders six “coherent actions” that can move Makassar to a healthier, “*Sombere* (kind-hearted and hospitable) and Smart City.” BHC has been working with the city planning office to incorporate the map findings into its bottom-up planning processes and the 5-year mid-term plan for Makassar.

## Introduction

Municipal planning often requires coordination of priorities and funding across many levels of government. In some cases, coordination is organized from the bottom up, meaning citizen feedback is gathered and filtered upward, sometimes to the national level [1]. In other cases, planning is done from the top down, where national priorities are filtered down to the city along with conditional funding from the top level. Sometimes a country combines both approaches for a mixed system. In all cases, city planners face a wide range of constraints and complexities when prioritizing what will be funded for their particular context, and the result is often that key activities are left out or left unfunded. The United States Agency for International Development (USAID) funded Building Healthy Cities project (BHC) partnered with four Asian Smart Cities beginning in 2017 to test ways to more effectively define city plans multi-sectorally with the goal of better health outcomes, or healthy city planning. This paper explores BHC’s systems mapping approach in one partner city, Makassar, Indonesia, where it has proved to be an effective tool for prioritizing activities and supporting partnerships within a mixed bottom-up/top-down planning system.

Makassar is the fifth largest city in Indonesia, with a population of 1.4 million as of 2020 [2] that is growing rapidly [3]. The city is a trading center and is the provincial capital of South Sulawesi. Makassar launched a Smart City technical team in 2017 [4]. The objective of the Smart City program is to promote cities that improve the quality of life of its citizens through infrastructure, a clean and sustainable environment, and the application of “smart” solutions, including information and communications technology. These objectives are expected to have positive effects on the health of Makassar’s residents.

Tobacco smoking is a leading risk factor for ill health in Makassar. According to the 2018 National Health Research Study (RISKESDAS), 25% of the population in Makassar were smokers; of those, most (98%) were men. In addition, 31% of the population was exposed to a passive smoker in the same room on a daily basis [5]. The most common infectious diseases in Makassar as of 2019 include tuberculosis (332/100,000 population), and dengue (17.8/100,000 population). That same year, the prevalence of pneumonia among children 0–5 years in Makassar City was 10% [5]. Low income residents of the city are often at greater risk for these conditions [6].

Barriers to data use have affected planning for and management of these urban health issues in Makassar. At the start of BHC in 2017, there was no universal policy in place that required every department to share its data with the Smart City data dashboard, a central, real-time data center which is nicknamed the “war room” [7]. Each department developed its own data sharing policies, and therefore very different processes for accessing data. Departments most often used Excel to collect and process data, and lacked data analysts with the skillset needed to conduct detailed data analysis, resulting in only cursory analysis and data visualization [8].

These complex issues make it difficult to prioritize health-related activities during city planning, which in Makassar is centered on the annual *Musrenbang*, a mixed planning process that includes a large bottom-up component that starts at the sub-district level [9]. Musrenbang requires a complex set of planning criteria and creates a long list of potential activities that city planners must synthesize and prioritize. The list is then weighted against other top-down priorities such as the Sustainable Development Goals, and mid-term and long-term goals from the provincial and central governments. This process requires a systematic approach to analyze problems and develop efficient and effective programming. BHC’s systems mapping approach is an effective tool that organizations and cities can use to help with this planning process in order to effectively address adaptive challenges in complex environments.

Before we describe this process, its findings, and its use in Makassar, it is worth noting other systems approaches that have been tried in Indonesia. In Bandung, West Java, collaborative planning has been implemented to involve multiple sectors and communities in the *rembug bersama* (gather and discuss) process, to formulate, implement, and evaluate programs. The aim is to integrate all members of the community in program development [10]. Within Makassar itself, some prioritization exercises have been attempted in the last four years since the Smart City program began in 2015. As part of the Smart City Master Plan, every city government department proposed innovative programs to address problems within Makassar [11]. As outlined by the Smart City Plan framework, the driver for all components of the work is meant to be ICT capacity, capability, governance, and infrastructure. For example, the multiple sectors involved in health, led by the Makassar City Regional Planning and Development Agency (Bappeda), gathered and discussed health priorities and innovations. The results were recorded into the Musrenbang process through the SIPPD, an information system for city planning and development.

The Smart City effort builds upon earlier initiatives. Makassar adopted a “Healthy City” effort as far back as 2007, which established a multi-sector forum and a workplan for Healthy City Makassar [12–16]. While the city was able to utilize this initiative to win the Swasti Saba Wistara award in 2011, review of this effort found that as of 2015 implementation of the workplan had been stymied by single sector thinking, lack of dedicated budget, and misunderstanding of what Healthy City meant among municipal decision-makers. By 2020, evaluations of the Healthy City effort found that training and socialization of the concept had improved (though may not have always been effective), and that it had also increased women’s engagement and political commitment [12–16]. This effort also led to the Sombere City initiative, which is meant to complement Smart City efforts with “heartware,” or strengthening the social and organizational context [17]. KOTAKU, or City without slums, was another national program that started in 2018 in Makassar to address multi-sector causes of poor living conditions in poorer areas of the city [18–20].

In BHC’s initial discussions with Kominfo, Bappeda, and the Mayor’s office in 2018, we heard that what the city still needed was additional support for both convening of the regular multi-sectoral Healthy City Forum coordination meeting, and a way to collect, store and update the data for the 170 Healthy City indicators. The regular meeting schedule had been disrupted by frequent turnover of staff, and without data they could not effectively track or fully implement the Healthy City strategies.

## Methods

The full methodology used for this work is defined in another article in this issue, Pomeroy-Stevens et al. (2022) [21]. In summary, BHC’s mapping methodology is based on qualitative data gathered during baseline assessments [6, 8, 22], which were then discussed at participatory workshops (including a series of focus group discussions) and town halls, as described in Table 1, which also provides the total participants for each phase.

**Table 1:** Summary of Makassar’s systems mapping process

Step	Dates	Source of data used to facilitate workshop	Participants	Data analysis
Defining Context	13–14 September 2018	Analysis of baseline assessment data	38 stakeholders	Cause-and-effect analysis to develop casual loops.
Finding Leverage	Leverage workshop: 30 April 2019	Context Map	42 stakeholders	Identify leverage opportunities and high impact actions.
	3 Town halls: (1) 22 February 2019 (2) 22 March 2019 (3) 21–27 June 2019	Relevant loops from Context Map	(1) Women’s group: 20 participants (2) Slum area group: 20 participants (3) Middle income group: 13 participants	Define leverage opportunities.
Creating Action	28–29 January 2020	Context Map and leverage opportunities	68 stakeholders	Identify a set of actions to move Makassar toward its long-term goal of being a world class city that is healthy and livable for all.

Source: For more details on these workshops, please see [23–25]

Sampling of participants was done purposively to achieve representation from all key sectors identified during the baseline assessments and as defined by our discussions with city leadership during the establishment of the project. This sampling cut across seven sectors (health, public works, public/urban planning, communications and informatics, environment, education, social services, and women’s empowerment) and included governmental, academic, nongovernmental organization, civil society, private sector, and donor respondents. For the citizen town halls, BHC worked through local civil society organizations to invite community members representing groups selected as most marginalized during the baseline health needs assessment. In total, the BHC Makassar team engaged 240 stakeholders over the course of this work from 2018 to 2022.

While systems maps can be developed from either qualitative or quantitative data, this study relied on qualitative methods for data collection, and the analysis used a grounded theory lens with the goal of revealing emergent patterns, relationships, and behaviors in the city system. Because the project had collected volumes of key informant interview and direct observation data under the three baseline assessments, these data were first re-analyzed in Excel to tease out key barriers and enablers under the themes uncovered under health needs, data use, and the political economy [26]. These were then presented as a launching point for focus group discussions in the first workshop, and a semi-structured list of questions was used to test the validity of the lists and then begin to develop loops in a participatory fashion to understand how these issues relate to each other. Each subsequent workshop facilitated participatory analysis to iterate the maps and develop the next level of findings. After each workshop, the study team then worked together to test the logic of the relationships within and across loops in order to build the causal loop diagrams in Kumu software, which was the primary vehicle for synthesizing the results [27].

Further details about the qualitative data analysis of the workshop data and visualization can be found in Pomeroy-Stevens et al. (2022) [21]. A unique aspect of the final maps are that they are available online via Kumu for anyone to view and navigate. While the software is not entirely free to the map developer (BHC does pay a workspace fee for each city map), the access to the final maps is free and openly available. Readers will find links to the Makassar maps throughout this article and are encouraged to review them for more detail.

## Results

Makassar’s Smart City program had a pre-existing goal statement which is “Sombere and Smart City.”^11^ Sombere means “kind-hearted” and identifies Makassar as a hospitable place to live. Reframing this goal statement as an aspirational “healthy and resilient city system” goal created the basis for the system’s Guiding Star and vision statement. The mutually agreed upon vision statement for Makassar was “Makassar as a world class city that is healthy and resilient for all.”

Building from this shared vision, the Makassar Context Map—available in an interactive format here (https://embed.kumu.io/2de1ef204cd9d98002558a87df17ebe3)—centers on a central “Deep Structure” called “Innovation Stagnation.”

This Deep Structure synthesizes feedback that while Makassar government and leaders have been actively innovating and utilizing new technology to expand and enhance city improvement efforts, this has actually increased the strain on existing infrastructure, because it has sped up the pace of development and attracted new people to the city. This increased strain has decreased the ability to achieve positive results in the city. The existing infrastructure and workforce simply do not have the capacity to keep up with the increasing need created by this drive toward innovation. An expansion of this foundation is necessary to effectively implement and sustain the city’s vision for growth and achieve positive outcomes. When the community recognizes that city improvement initiatives are not achieving the promised result, citizens are less likely to engage and support their implementation. Without the backing of the community, government efforts to bring innovative approaches to advancing healthy development are undermined.

Every interconnected factor in the Context Map is part of a loop that reveals a pattern within the city system. The Deep Structure is expanded upon by 32 feedback loops that are categorized into four map areas: (1) leadership, governance, and financing; (2) infrastructure and workforce; (3) collaboration and data; and (4) community cohesion and awareness. Table 2 summarizes these areas.

**Table 2:** Makassar context map areas

Areas	Description
Leadership, governance, and financing	Any new actions to help Makassar grow in a healthy way will need to work with the behavior, knowledge, and practices related to accountability and management in the governance structures, which are defining, developing, and enforcing policies, procedures, rules, guidelines, etc., and continually monitoring their proper implementation. Community participation in budgeting processes is limited due to limited access to the information to monitor, evaluate, and audit the process, and to challenge decisions.
Infrastructure and workforce	Any forward planning by the city administration needs to ensure that basic needs of the citizens are being planned and provided for in an equitable way for healthy growth and development of Makassar City. Having an enabling infrastructure and environment, and a trained workforce to provide essential services such as health, education, and sanitation, is absolutely necessary for citizens to survive.
Collaboration and data	Any city, in order to grow in a smart way, needs to have a well-established information system. Information systems are an integrated set of components for collecting, storing, and processing data for providing information. The purpose of these information systems is to turn raw data into useful information that can provide evidence and correct knowledge for decision-making.
Community cohesion and awareness	Community awareness is the community’s understanding of the importance and implications of various programs, policies, and laws developed for them. The ability to engage the population in healthy development efforts is recognized as the key to achieving program impact goals and enabling strong leadership.

To continue participatory analysis, BHC facilitated a gathering of city stakeholders to incorporate priorities identified through the Musrenbang process into the map. As a result, we identified the key obstacles and opportunities that will need to be addressed to achieve the vision statement of a healthier Makassar. These details can be found in the Kumu maps, and we share the implications in the discussion section.

Leveraging of the Context Map revealed which changes might have the biggest impact, and what parts of the context may never change. Based on this analysis, and feedback and information received from the stakeholders, BHC identified three leverage opportunities, or areas where change would have the biggest impact. These are described in Table 3 and illustrated in the interactive Leverage Map (https://embed.kumu.io/00d0112de9a758e29482f6f854e35b07).

**Table 3:** Three leverage opportunities

Leverage opportunity	Description
1. Increasing quality, accessibility, and timeliness of data for decision-making	This leverage opportunity is, at its core, about enhancing and expanding data driven decision-making. If key stakeholders have good quality data for decision-making, the city will be able to effectively target and distribute resources to achieve positive results.
2. Ensuring equity and transparency in policy implementation to ensure equitable community access to services	Policy can improve progress on achieving positive goals by providing clear tools, methods, and expectations for fostering community participation and effectively managing and utilizing data. By extending transparency of policy and practice to community members, they will be able to develop realistic expectations for government services and feel empowered to support accountability.
3. Maximizing community participation in programs to improve community awareness of health promoting practices and resources	In order to improve access for all, it is necessary to improve government outreach and increase citizen education, awareness, and participation in city planning efforts. If inequality is reduced and community participation is increased, there will be greater acceptability and participation in existing city processes and less exclusion/higher utilization of services.

The obstacles and opportunities were discussed at BHC’s Theory of Action workshop held in Makassar. Using the system leverage opportunities and the four main areas of the Context Map, BHC and stakeholders developed a set of suggested coherent actions to move Makassar toward its vision statement*.* The actions are detailed in Table 4 and include: sustaining municipal support for the goal of a healthy Makassar; leading the way on a circular economy; creating a culture of data for health; creating a more water-resilient city; growing a healthier next generation of citizens; and encouraging healthy lifestyles for non-communicable disease prevention.

**Table 4:** Proposed Makassar Healthy City actions

How might we	Proposed coherent action summary
Sustaining municipal support for the goal of a healthy Makassar	
Foster meaningful accountability, increase communication and coordination, and make policy decisions and processes more transparent and participatory?	The purpose of this action is to foster meaningful dialogue, increase communication and coordination, and make policy decisions and processes more transparent and participatory to sustain a whole-city health Makassar effort. This would happen by using information and communications technology to increase transparency in the Musrenbang process and engaging city officials in the RT/RW (sub-district or kelurahan) or neighborhood levels. This would create not only a comprehensive approach to building healthier communities but also opportunities to strengthen community participation.
Sustain a whole-city healthy Makassar effort?	
Leading the way on a circular economy	
Address waste removal more effectively, even as our population grows?	The purpose of this activity is to create a new waste management economy. This would happen by engaging the private sector, nongovernmental organizations, and communities affected by poor waste management and its health effects to carry out innovations in waste reduction in a revenue generating way. This model realizes that environmental management is not just the responsibility of the government.
Creating a culture of data for health	
Make it easier to use data to support health in Makassar?	The purpose of this activity is to improve the timeliness and usability of data across sectors relating to urban health. This would include building the capacity of health workers and government officials, and using existing technology and resources such as DHIS-2 and the war room. This would create a trained staff, improve data management, reduce the manual process of data entry, and promote data utilization across sectors.
Encourage and build the capacity of health care workers and government staff to collect, manage, and use data?	
Strengthen the existing data systems?	
Creating a more water-resilient city	
Keep producing enough clean water for a growing population even while waste and flood waters keep rising?	The purpose of this activity is to create resiliency to rising flood and waste water in a sustainable manner. This would happen by engaging multi-sector stakeholder partnerships including government, nongovernmental organizations, private sector, and those communities most at risk of flooding. This would create a safe, healthy, and livable environment for all sections of society.
Unclog waterways and drains while also addressing the need for more job opportunities in informal settlements?	
Growing a healthier next generation of citizens	
Create a city that promotes heathier children?	The purpose of this action is to build Makassar into a child friendly city by keeping the focus on health and living environments of children through a bottom-up approach, active community participation, and multi-sector engagement. This would reduce inequities from birth, increase women's ability to participate in the workforce, and lower health care costs in the long term.
Ensure access to healthy food for every Makassarese?	
Ensure awareness about healthy food and hygiene among food handlers and citizens?	
Encourage children to adopt health promoting behaviors?	
Encouraging healthy lifestyles for noncommunicable disease prevention	
Reduce the risk of noncommunicable diseases within communities while also greening our living spaces?	The purpose of this action is to improve healthy lifestyles to reduce noncommunicable disease. This would happen by strengthening community-based intervention for noncommunicable disease prevention. This model will encourage community-based intervention by optimizing operational incentives and strengthening healthy ecosystems.

BHC used a refining process described in another article in this issue, Pomeroy-Stevens et al. (2022) [21], to flesh out these actions and break them down into sub-actions based on the leverage opportunities and map areas. An example of this breakdown for action #1 “sustaining municipal support for the goal of a healthy Makassar” can be seen in Table 5.

**Table 5:** Coherent action example—sustaining municipal support for the goal of a healthy Makassar

Area	Leverage opportunity	Sub-action
Leadership, governance, and financing	Data for decision-making	Bappeda and the Department of Communication and Information (Kominfo) should decide on a regular multi-sector mechanism for discussing cross-sector data alerts that come up in the war room, to build in the expectation that these data will be regularly discussed and used for planning.
	Equitable policy and leadership	Develop municipal policies, workforce training programs, and technical support to increase not just data reporting but also data usage and visualization within the war room by city leadership, with a particular focus on getting the related sector data used as part of regular city mid-term, near-term, and long-term planning.
		Bappeda should regularly work across donors/nongovernmental organizations/private sector on key coherent action areas to ensure they are aligning funding and programs with Healthy Makassar goals, and targeting alleys (neighborhoods) with highest needs.
	Citizen engagement	Use a systems approach for the Musrenbang that best utilizes citizen feedback in the process to develop a budget and workplan each year. The method used should be transparent, systematic, and replicable, and allow for public release of the findings
Infrastructure and workforce	Data for decision-making	Improve workforce capacity for using data through changes to university programs for each sector, civil servants training courses, or some other form of regular capacity development.
	Equitable policy and leadership	Improve government investment for facilities and infrastructure to support data management and encourage data-driven decision-making. This investment needs to be supported by enhancing workforce capacity for data managers to meet the need for qualified workforce to use information technology and manage data from collection to utilization.
	Citizen engagement	Strengthen the integrated system for managing citizen complaints with GIS technology into the central war room that is accessible for the communities to track their complaints and progress and to give feedback.
Collaboration and data	Data for decision-making	Bappeda and Kominfo should collaborate to use collected data from communication channels including citizen reporting systems, social media, and electronic-based channels to identify gaps in service and inform service delivery policy as well as for program planning and budgeting. These data can be used for bottom-up planning during the Musrenbang.
	Equitable policy and leadership	Encourage a "One Data" regulation plan to be ratified at the city level. In addition, the city government should implement a city level policy that requires all government departments, nongovernmental organizations, and private sectors to share their data including mechanism, type of data, variable, data use for decision-making, person in charge, and their incentive. A one data governance model can be implemented across different systems to cover many data sources and departments which involve multi-sector stakeholders in the process of data integration and dashboard management.
	Citizen engagement	Increase outreach campaigns on key environmental health and public health topics. Citizen reporting system data can be used to identify the topics that are most pressing, such as road safety, opening drains by reducing loose garbage, etc. These campaigns can be timed to coincide with key points in the Musrenbang process to ensure citizens know what to ask for to fix any enduring health-related issues in their neighborhoods.
Community cohesion and awareness	Data for decision-making	Increase transparency of the decision-making process and how selected programs were implemented. This can be done via public posting of Musrenbang rankings/ratings.
	Equitable policy and leadership	Develop and use a city monitoring and evaluation plan for monitoring progress on the "Sombere and Smart City" goals that are tied to RT/RW (sub-district or kelurahan) level improvements.
	Citizen engagement	Engage Healthy Alleys to do their own prioritization exercises around what they need to improve their Healthy Alley indicators, so that they come prepared to ask for these items during the Musrenbang.
		Review the Musrenbang process to see whether it can be augmented by developing a year-round community collaboration platform that supports standardized, trusted, and ongoing community engagement.

The resulting Action Plan underwent a validation process in 2021 and 2022, which has seen several unexpected and positive extensions of this work to support city planning processes. This included support to develop the Musrenbang prioritization plan for 2021 and 2022, and the mid-term city planning for 2021–2026 [28].

## Discussion

Systems thinking has proven to be a useful tool for understanding complex challenges, and in particular, for helping to build partnerships for tackling those challenges. Palutturi (2013), in his assessment of the Healthy City Makassar movement, noted key factors that could challenge effective partnerships [15]. These are shown in Table 6 below. Based on this rubric, we can see where BHC’s systems approach has been able to contribute to stronger partnerships for supporting both Healthy City Makassar and the Smart City initiative. These areas relate to clear communication, purpose, and resources.

**Table 6:** Key constraining factors to effective healthy city implementation (Palutturi, 2013)

Environment/context	Clear communication
Enabling climate	Open and frequent
	Established formal and informal communication links
Membership nature	
Commitment and synergy	Purposes
Understanding the partner needs and trust	Concrete goals and objectives
Collaborative members seeing collaboration as in their self interest	Shared vision
Voluntarism relating to members' contribution	Shared responsibilities, benefits, and risks
Process and structure	Resources
Clear roles and responsibilities	Strong leadership and political will
Community participation	Skills and capacity

Source: Palutturi S. Healthy Cities Implementation in Indonesia: Challenges and Determinants of Successful Partnership Development at Local Government Level. Griffith University; 2013.

Clear communication is a key part of successful urban health interventions, as the determinants are complex and sometimes hard to understand. The BHC workshops provided a starting point for formal communications, which helped to also revive interest in attendance of the Healthy City Forum meeting by designated departments, which then provided further avenues for informal communications. While COVID-19 did slow the convening of these meetings in 2020–2021, BHC held one-on-one meetings with member departments to keep momentum toward the goals set during the systems mapping sessions. Through Bappeda’s engagement with the BHC process, they were able to mobilize several private sector and civil society organizations on key topics identified during the mapping, and they worked through the Healthy City Forum to secure commitment charters to tackle both wastewater management and nutrition. One shortcoming of BHC’s process was that we were not able to extend this process all the way to the sub-district and village healthy city working groups, which were identified as part of the Healthy City coordination structure [29].

Purpose of partnerships is where we felt the BHC systems approach brought the most added value. As noted in the results, creating a shared vision at the beginning of the workshops is a simple but crucial step to ensure similar motivation and goals for all involved. The final result of the process is a mutually defined Action Plan which provides a roadmap for Makassar stakeholders to achieve their vision for a healthy city. For each action, the plan provides recommended roles and responsibilities, an estimate of the implementation costs, suggested nongovernmental partners, monitoring and evaluation indicators (based on the Healthy City indicators), and finally an estimate of the potential impact of these combined actions on key health outcomes. By basing the plan on a participatory systems mapping process, it bypassed concerns about how context appropriate the plan is, and had widespread buy-in for the actions even before the plan was finalized. The systems maps provide a publicly available, transparent visual framework that explains why these actions were chosen. The maps are in the process of being handed over to Bappeda for their ongoing use even after BHC ends.

Finally, regarding resources, while BHC as an outside organization cannot change staffing norms, we have tried to buffer the leadership of work toward a healthier Makassar against frequent staffing changes on both the forum and in political leadership by broadening the support and ownership of this work. By engaging with Kominfo as leadership for Smart City, Bappeda as leadership for regular financing and planning, and the Mayor's office, this can help keep momentum for the work going even as individuals rotate in and out of positions.

Finally, as noted in Bachani et al. (2022), the systems mapping process has provided a new skillset and capacity for prioritization to the city [30]. The results of the systems mapping helped to define all of the interacting factors that might sabotage a successful knowledge management system where the Healthy City achievements and data will be stored and routinely updated. Using this more nuanced understanding of the context, BHC was able to support multi-year development of a multi-sectoral data system that includes all 170 Healthy City indicators and has strengthened data operator skillsets for data use and visualization [31]. These indicators have been integrated into the system called “SIAGANG,” meaning “together” in the local language (Sistem InformAsi proGram kota sehAt yang iNklusif dan teriNteGrasi / Inclusive and Integrated Information System for Healthy City) [32]. Without the mapping we would have missed key capacity building steps around data ownership, sharing, and trust; mutual accountability; data quality; and need for systems integration. Further training of urban health leadership cadres on the systems approach would be useful to create a pipeline of staff who can plan for complexity [33].

There were unexpected benefits of using this process to develop the Action Plan. First was the uptake of the systems mapping methodology by Bappeda to support the Musrenbang prioritization process to develop city planning for 2021 and 2022. Musrenbang is conducted annually, and citizens from the sub-district up must articulate their needs to the local government in hopes of directing funding to their neighborhoods. Bappeda must then sort and prioritize those needs, determine what programs will be funded, and align them with top-down priorities, often in a very compressed timeframe. Bappeda requested that BHC facilitate trainings on using a systems approach to prioritize high impact programs suggested through the Musrenbang process, to support efforts to increase the efficiency of multi-sector urban spending [34]. The BHC systems map approach allowed Bappeda to break down the complex issues raised during Musrenbang. BHC trained Bappeda on how to use the leverage opportunities that BHC identified in the map for prioritization, which eased the process of identifying high impact actions for the city prioritization plan for 2021 and 2022. In addition, a June 2021 workshop cohosted with Bappeda showed the utility of this methodology for guiding mid-term city planning for 2021–2026 [28]. During the workshop, Bappeda and 14 city government departments used the BHC-recommended Action Plan and the BHC-identified leverage opportunities as a framework to develop high impact actions that also addressed the national and provincial priorities. The Action Plan was a useful tool for helping to identify the city’s priorities, as the BHC map was a result of a comprehensive problem analysis of Makassar City.

Despite the successes so far, it is important to note some constraints and limitations. Due to human resources constraints and, later in the process, COVID-19 restrictions, the bulk of the community engagement on the maps happened between the Context Map development and leveraging phase. Some community groups were not engaged, including disabled persons associations, and communities in remote areas of the city. Ideally, BHC would have re-engaged all key demographics immediately after the Theory of Action workshop. With COVID-19 restrictions now easing, BHC is aiming to re-engage some communities before finalization of the plan in early 2022. Another caveat to the systems mapping approach is that, as with any qualitative data collection, the results are only as complete as the stakeholders interviewed. We acknowledge that we had less private sector engagement than desired, and this is something that the city should consider as they move forward with the actions. In addition, while BHC did interview refugees and migrants as part of the baseline data that was used for the Context Map, we were not able to socialize the map with those particular groups. Finally, the Action Plan is not meant to be a comprehensive list of every needed action to improve the city system, but rather those actions that are at the intersection of the key leverage opportunities and what is most feasible for the city to address without changing what is “frozen” in the map.

These limitations noted, BHC’s systems approach offers several improvements in action planning over some more traditional methods of consensus-based policy development. In Makassar City, Bappeda is a leading sector in developing both annual and mid-term city plans. In the past, these plans have been developed in part by using a logical framework, where every department proposes programs to be included [35]. The BHC systems approach encouraged Bappeda to develop the plans in a more integrated way; all departments gathered together and held a comprehensive discussion to identify the problems, key opportunities, and high impact actions.

Some lessons that might be useful to other cities that are considering using this process are how this process encouraged multi-sectoral discussion, and in some ways reduced territoriality regarding sector resources by looking at patterns that effected all sectors equally. It was also helpful to define sector-specific roles and responsibilities for each high impact action, with one department, such as Bappeda, providing overarching leadership, guidance, and coordination. Finally, the open source nature of Kumu, the interactive map used to create the maps, eased data sharing and use issues—it can easily be transitioned to a different owner such as the government, nongovernmental organizations, and private sector partners, and is publicly available for anyone who wants to view the map.

## Conclusion

The systems approach is a promising option for cities looking to conduct comprehensive, multi-sector planning and budgeting. The city of Makassar successfully applied this approach to their mid-term city planning for 2021–2026, which allowed them to identify comprehensive problems with a broader view using the systems map, and improve partnerships across multiple sectors to design coherent actions as solutions. Makassar City, through Bappeda, should expand this approach to be used by all departments for their planning. Other cities can benefit from Makassar’s current and future experience introducing a new systems approach.

## Data Availability

The resulting Context (https://embed.kumu.io/2de1ef204cd9d98002558a87df17ebe3) and Leverage Maps (https://embed.kumu.io/00d0112de9a758e29482f6f854e35b07) are available on the open-source platform Kumu.
